# A Soft Wearable and Fully-Textile Piezoresistive Sensor for Plantar Pressure Capturing

**DOI:** 10.3390/mi12020110

**Published:** 2021-01-22

**Authors:** Yongsong Tan, Kamen Ivanov, Zhanyong Mei, Hui Li, Huihui Li, Ludwig Lubich, Chaoxia Wang, Lei Wang

**Affiliations:** 1 Shenzhen Key Laboratory for Low-cost Healthcare, Shenzhen Institutes of Advanced Technology, Chinese Academy of Sciences, 1068 Xueyuan Avenue, Shenzhen University Town, Shenzhen 518055, China; ladtys@outlook.com (Y.T.); kamen@siat.ac.cn (K.I.); hui.li1@siat.ac.cn (H.L.); hh.li@siat.ac.cn (H.L.); 2 Key Laboratory of Eco-Textile, Ministry of Education, College of Textile Science and Engineering, Jiangnan University, 1800 Lihu Road, Wuxi 214122, China; 3 Institute of Biophysics and Biomedical Engineering, Bulgarian Academy of Sciences, Acad. G. Bonchev Str. Bl 105, 1113 Sofia, Bulgaria; 4 College of Cyber Security, Chengdu University of Technology, Chengdu 610059, China; meizhanyong2014@cdut.edu.cn; 5 Faculty of Telecommunications, Technical University of Sofia, 1000 Sofia, Bulgaria; lvl@tu-sofia.bg

**Keywords:** textile piezoresistive sensor (TPRS), rGO-cotton fabric electrode, Ag fabric circuit electrode, insole, dynamic plantar pressure

## Abstract

The trends of wearable health monitoring systems have led to growing demands for gait-capturing devices. However, comfortability and durability under repeated stress are still challenging to achieve in existing sensor-enabled footwear. Herein, a flexible textile piezoresistive sensor (TPRS) consisting of a reduced graphene oxide (rGO)-cotton) fabric electrode and an Ag fabric circuit electrode is proposed. Based on the mechanical and electrical properties of the two fabric electrodes, the TPRS exhibits superior sensing performance, with a high sensitivity of 3.96 kPa-1 in the lower pressure range of 0–36 kPa, wide force range (0–100 kPa), fast response time (170 ms), remarkable durability stability (1000 cycles) and detection ability in different pressures ranges. For the prac-tical application of capturing plantar pressure, six TPRSs were mounted on a flexible printed circuit board and integrated into an insole. The dynamic plantar pressure distribution during walking was derived in the form of pressure maps. The proposed fully-textile piezoresistive sensor is a strong candidate for next-generation plantar pressure wearable monitoring devices.

## 1. Introduction

The flexible wearable devices, an unobtrusive sensing interface with the human body, have already become a key tool for capturing human physiological parameters [1,2,3,4]. Recently, developing flexible wearable devices has focused on improving the softness, breathability and stability [5]. Textiles have become an ideal substrate material for flexible wearable devices due to their high extensibility, easy processing, and low cost [6]. Cotton textiles with large contact surfaces and excellent mechanical properties were introduced to designs for flexible sensing systems that are comfortable for the wearer [7]. Their ad-vantages are essential when capturing human information through a fully-wearable monitoring device. Thus, a significant number of flexible textile devices with high sensitivity and biocompatibility with skin have been designed for capturing human movement [8,9,10,11]. Piezoresistive pressure sensors possess tremendous potential for wearable device applications due to their excellent sensitivity, durability and biocompatibility with human skin [12,13,14,15,16,17]. In these sensors, the external pressure stimuli induce a change in resistance, that can be measured and utilized as feedback about the information state of the human body. For instance, an ultrasensitive fiber-based piezoresistive sensor that can be used to observe the walking signal was fabricated [18].

The wearable plantar pressure capturing systems can greatly benefit from the textile piezoresistive pressure sensors. Analysis of dynamic plantar pressure patterns is used for early warning alarm and prevention of foot deformities, or discovery and rehabilitation monitoring in the advanced stages [19,20]. The timely detection of abnormal pressures can prevent the development of diabetes ulcers [21,22]. Additionally, deviations in gait dynamics could indicate the progression of dementia development [23]. Traditional sys-tems for capturing plantar pressure include force plates and pressure sensing walkways [24]. However, the use of these devices is limited to laboratory settings, which makes them unsuitable for continuous monitoring [25,26,27,28]. Hence, active engineering and research ef-forts have been devoted to realizing continuous capture of plantar pressure in natural set-tings. Sensing insoles can detect dynamic pressure patterns under the main weight-bearing locations of the foot [29,30,31,32]. Even though these systems allow sensing with sufficient accuracy, many of them are not biocompatible with the human skin and easily deform under repeated external pressure, which restricts their application in gait monitoring [33,34,35]. Thus, the trends in smart footwear design are to address the aspects of reliability of signal capture, biocompatibility, and comfort of wearing.

In the present work, we aimed to design a flexible, yet mechanically and electrically stable, fully-textile piezoresistive sensing device for integration into a monitoring system to capture plantar pressure. Here, cotton fabric, a basic wearable material characterized by softness, breathability, and high conformability, is designed as the substrate for con-structing the fabric electrode. The reduced graphene oxide (rGO)-cotton fabric electrode and Ag fabric circuit electrode demonstrate a high conductivity of 98.3 kΩ/cm and 0.3 Ω/cm, respectively. Thanks to the use of rGO and Ag, the proposed textile piezoresistive sensor (TPRS) has achieved high flexibility and durability, high sensitivity of 3.96 kPa^−1^ in the lower pressure range of 0–36 kPa, a wide force range (0–100 kPa), a fast response time (170 ms) and remarkable stability of parameters under repeated stress (1000 cycles). To illustrate the application of TPRS in plantar pressure capture, six TPRSs were mounted on a flexible printed circuit board attached to a textile insole to form a complete sensor device for monitoring plantar information. The device was connected to a custom electronic acquisition module for reflecting plantar pressure. The results of the gait signal capturing and pressure distribution show that the textile flexible piezoresistive sensor has a huge potential for application in sensing footwear.

## 2. Materials and Methods

### 2.1. Materials and Reagents

The graphene oxide (GO) was purchased from Suzhou Crystal Silicon Electronic & Technology Co, Ltd., Suzhou, China. The Ag paste and sodium hyposulfite were pur-chased from Aladdin. All reagents were of analytical grade and were used as received without further purification.

### 2.2. Fabrication of rGO-Cotton Fabric Electrode

First, the cotton fabric was treated with oxygen plasma to enhance the interface adsorption. It was then cleaned with deionized water to remove the impurities; after that, the clean fabric was treated again with oxygen plasma for 5 min. Next, the rGO cotton fabric electrode was prepared by repeated impregnation and reduction. The fabric was immersed in 3 mg/mL GO deionized water solution for 15 min and dried in an oven at 80 °C for 30 min. After repeating this process three times, the GO on the surface of the cotton was reduced to rGO in sodium hyposulfite solution (10 g/L) at 90 °C for 8 h.

### 2.3. Fabrication of Ag Fabric Circuit Electrode

A mask was formed in the polyimide (PI) tape covering the surface of the cotton fibres using a laser-cutting machine. The unnecessary cut part of the PI tape was removed using tweezers to obtain the fabric electrode circuit. After treatment with oxygen plasma, the circuit surface was coated with silver paste, and the conductive circuit was treated in a vacuum drying oven at 70 °C for 30 min. Then, the PI tape was removed.

### 2.4. Fabrication of the Textile Piezoresistive Sensor

The TPRS was constructed using the conductive rGO cotton fabric electrode and Ag fabric circuit electrode, where the outer surfaces of both were covered by a polyimide tape substrate. In addition, the Ag fabric circuit electrode was electrically connected with an embedded copper electrode in the role of an interconnector.

### 2.5. Fabrication of the Sensor Insole

With a limited number of sensors, the proper selection of sensor positions under the foot is essential [36]. Six piezoresistive sensors were mounted under the main weight-bearing positions in the insole, namely the big toe, first and fourth metatarsal heads, the midfoot, and the heel. The sensors were first mechanically fixed to the flexible printed circuit board using thin double-sided adhesive tape, and then were electrically connected. The sensor surface of the sole was covered with a thin transparent plastic sheet layer that was also sewn to the textile base of a commercial insole. Finally, the complete sensor insole was placed into shoes and connected to the electronic acquisition module.

In order to test the accuracy in reflecting the pressure dynamic patterns, two commercial reference sensors have been introduced for comparison with TPRS during walking. Each reference sensor was carefully aligned on the top surface of its corresponding TPRS.

### 2.6. Characterizations

A scanning electron microscope (SEM) was used to investigate the morphology of the fabric electrode. The swatches were coated with a 5–10 nm Au layer before the SEM imaging. A Keithley 2400 source/meter (4-wire) was employed to measure the resistance of the rGO cotton fabric electrode and Ag fabric circuit electrode. The pressure was applied to the TPRS using a universal testing machine, and the resistance of TPRS was also tested using the Keithley 2400 source/meter (4-wire). Before the pressure testing of TPRS, a cube (10 mm × 10 mm) was placed on the contact area.

## 3. Results

**Figure 1:**
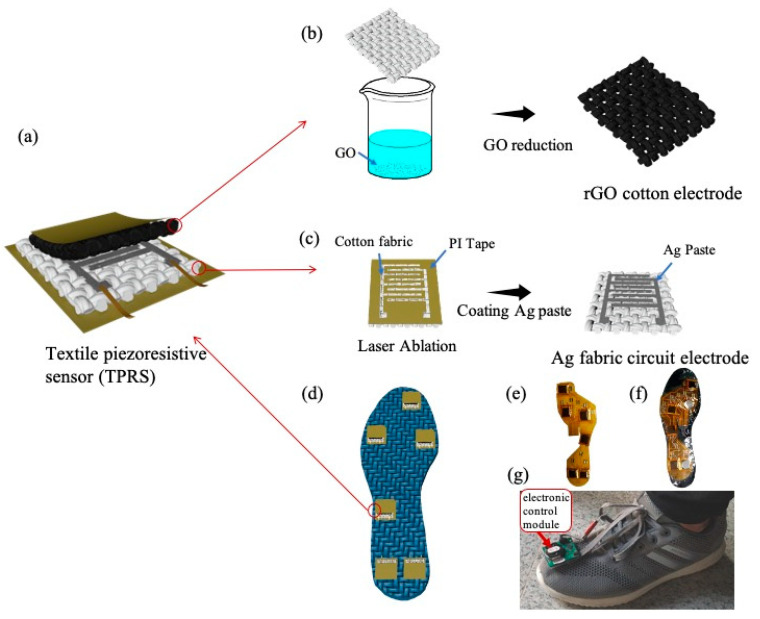
Schematic illustration of fabric electrodes and textile piezoresistive sensor (TPRS): (**a**) the preparation process of the fabric electrode coated with silver paste; (**b**) the preparation process of the fabric electrode coated with reduced graphene oxide (rGO); (**c**) the assembly process with Polyimide (PI) tape; (**d**) the preparation process of the complete TPRS, (**e**) flexible printed circuit board with attached TPRSs, (**f**) encapsulated insole and (**g**) in-shoe setup.

Figure 1 illustrates the fabrication process and structure of TPRS, as explained in the previous section. The TPRS comprises the conductive rGO-cotton fabric electrode (Figure 1b) and the Ag fabric circuit electrode (Figure 1c), in which the outer surfaces of the both were covered by PI tape substrate, as shown in Figure 1a. Further, the TPRSs were affixed in different positions on the insole to monitor the plantar pressure information, as shown in Figure 1d. Figure 1e–g show the flexible printed circuit board with sensors mounted on it, the encapsulated insole, and the complete in-shoe setup.

**Figure 2:**
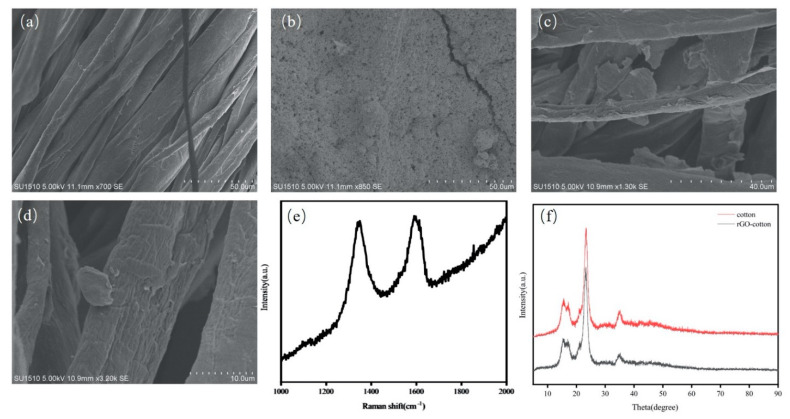
The scanning electron microscope (SEM) images of the surface morphology of (**a**) control cotton fabric, (**b**) the Ag fabric circuit electrode, and (**c**,**d**) the rGO-cotton electrode. (**e**) Raman spectrum of rGO-cotton. (**f**) X-ray diffraction (XRD) spectrum of cotton and rGO-cotton.

Figure 2 shows the SEM images of the morphology of the rGO-cotton electrode and the Ag fabric circuit electrode. Compared with the control cotton fabric (Figure 2a), a uniform coating layer of silver paste was formed on the surface of the cotton fabric, as shown in Figure 2b. The surface resistance of the Ag fabric circuit electrode was 0.3 Ω/cm (Figure S1a), which can be attributed to the excellent conductivity of the silver paste. Notably, the rGO is wrapped on the surface on the surface of cotton yarns, as shown in Figure 2c-d. The Raman spectroscopy is widely used to exhibit D and G bands from the graphitic structure (Figure 2e). The result shows that the D peak in rGO-cotton appeared at 1350 cm^−1^, and the G peak appeared at 1593 cm^−1^. The oxygen-containing groups in GO are effectively removed after reduction. Figure 2f compares the XRD patterns of cotton and rGO-cotton fabric. The X-ray diffraction pattern of rGO-cotton electrode shows a major peak at 23.3° (002). The (001) crystal plane of GO disappears and the characteristic peak in rGO at higher 2θ coincides with that of cotton fabric. This can be explained by the fact that the oxygen functional groups disappear after reduction [37]. The surface resistance of rGO-cotton electrode is 98.3 KΩ/cm (Figure S1b). A uniform rGO is successfully obtained on the surface of cotton fabric.

The sensing mechanism of TPRS is explained by the change in the resistance of the contact between the rGO-cotton electrode and the Ag fabric circuit one upon the application of pressure over the outer sensor surfaces. Increasing pressure leads to the formation of small compressive deformations that enhance the contact between the two conductive fabrics and reduces the interlayer distance between them. Thus, the number of electrical pathways between the two electrodes increases. Upon initial contact between the two electrode parts, the resistance of the sensor decreases rapidly, while this decrease becomes gradual upon reaching full contact between the surfaces of the electrodes. This change in the sensor resistance is explained by the fact that the graphene sheets are stacked together to form a graphite-like bulk body, which accelerates charge hopping between the overlapping graphene islands [38]. When high pressure is applied to the TPRS, charge-hopping occurs between the overlapping graphene islands. After unloading the pressure, the TPRS recovers its initial shape, which results in a decrease in the contact area and fewer electrical pathways. The changes in resistance upon the application of different pressures were also thoroughly tested. 

**Figure 3:**
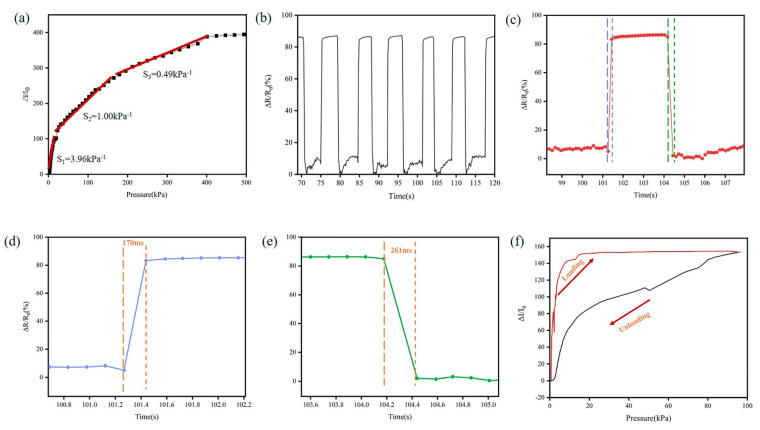
(**a**) The sensitivity performance of TPRS (**b**) real-time response of the sensor under an applied pressure of 5 kPa. (**c**) Pressure response showing a single cycle, and the corresponding (**d**) response time and (**e**) releasing time. (**f**) The hysteresis curves of TPRS.

In this study, the sensing performance of the TPRS is assessed in terms of normalized resistance change, response time, high durability and dynamic response. A computer-controlled force gauge platform and an electrical signal analyzer were used to test the dynamic characteristics under different test modes. To investigate the performance of TPFS, the relative current change (Δ*I*/*I*_0_) versus pressure is shown in Figure 3a. Additionally, the sensitivity of the TPFS is 3.96 kPa^−1^ in the lower pressure range of 0–36 kPa, while the sensitivity lowers to 0.49 kPa^−1^ in a higher-pressure range. This performance of TPFS in different pressure ranges is consistent with the results discussed above. Figure 3b shows the relative resistance change in a fully-textile piezoresistive sensor under a pressure of 5 kPa. In this case, the TPFS shows a stable resistance response, excellent repeatability and durability. The response and recovery times of TPRS were 170 and 261 ms, respectively, as shown in Figure 3d,e. Interestingly, the releasing time for higher pressure is much longer. Under a larger force, the rGO cotton fabric electrode and Ag fabric circuit electrode are in close contact, and recovery takes a longer time. Hysteresis is mainly caused by the interaction between the rGO cotton fabric electrode and Ag fabric circuit electrode. A low degree of hysteresis (*DH*) indicates lower hysteresis of the TPRS. The *DH* can be calculated by the following formula:

$$DH=\frac{A_{L}-A_{U}}{A_{L}}$$

where *A_L_* and *A_U_* are the areas under the loading and unloading response curves, respectively. The *DH* value of TPRS is 28.6%.

**Figure 4:**
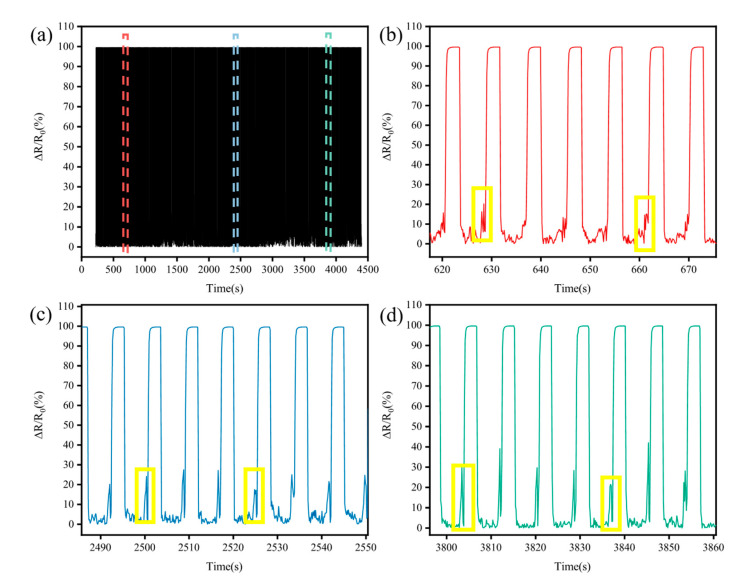
(**a**) The TPRS durability test (1000 cycles), (**b**) the enlarged view of the red area of (**a**), (**c**) the enlarged view of the blue area of (**a**), (**d**) the enlarged view of the green area of (**a**).

**Figure 5:**
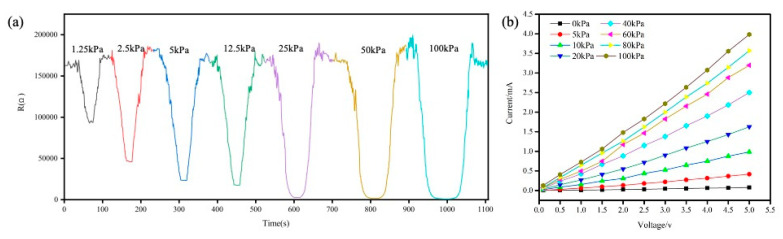
(**a**) Relative electrical resistance changes under different pressures (**b**) current-voltage characteristics.

In order to verify the adaptability of TPRS to varying plantar pressure and stability to repeated pressure cycles, cyclic force (including loading and unloading process) between 0 and 80 N at a speed of 20 mm/min with 5 s pause is applied to the TPRS. It is important for the TPRS to have excellent recovery characteristics to ensure a stable performance and a long lifetime. The durability performance along the repeated cycles is illustrated in Figure 4a. Due to the excellent recovery characteristics of the cotton fabric, the TPRS resistance returns to its initial value after unloading the pressure. The phenomena of signal drift and structural damage were not significant during the load/release cycles due to the excellent mechanical and electrical properties of the cotton fabric electrodes. As shown in Figure 4b-d, the resistance of TPFS fluctuated at the moment of loading and releasing the pressure. In the whole cycle, the inherent elasticity of the fabric electrode makes it difficult for the two fabric electrodes to respond quickly to the external pressure, which brings about a delay in resistance variation. Interestingly, we observe that the resistance of the TPFS has a jump, and then changes rapidly as the pressure increases, as shown in the yellow area of Figure 4b-d. This can be explained by the contact resistance changed between the rGO cotton fabric electrode and Ag fabric circuit electrode upon the application of pressure over the outer sensor surfaces. At the beginning, the resistance of the TPRS decreases suddenly when the two fabric electrodes first make contact. The increasing pressure leads to the formation of small compressive deformations that enhance the contact point between the two conductive fabrics and reduce the interlayer distance between them. For capturing plantar pressure, the sensor needs to have a high sensitivity and this ability was also tested, as shown in Figure 5a. It can be seen that the TPRS is more sensitive within a smaller pressure range, but less sensitive to pressure in a larger pressure range. This result is in good agreement with the above sensitivity, indicating that the TPRS is particularly sensitive below 50 kPa pressure. The TPRS is capable of operating up to the pressure of 800 kPa before failure (Figure S2). The results show that the TPRS can quickly feedback the pressure without damage, even under the condition of high pressure.

In order to interpret the relationship between current and voltage, pressure and current, and pressure and resistance, a circuit was designed. One of the benefits of the transimpedance amplifier circuit is that it maintains a constant voltage over the sensor, thus avoiding the influence of eventual non-linearities of the voltage-current characteristic of the sensor. As the reciprocal slope of the straight line represents the resistance of the TPFS based on *R*=*V*/*I*, the current and voltage of the sensor are in linear relationship when the resistance is constant. As shown in Figure 5b, the current increased almost linearly with the applied voltage and the slope of the line did not change at the same pressure. When the constant voltage was applied to the TPRS, the current through the sensor increased correspondingly with the pressure increase. The resistance will decrease when pressure is applied, resulting in an increase in the current (*I*=*V*/*R*). The slope of lines (*Slope*=*I*/*V*) will increase with the increase of pressure applied to TPFS. This indicates that the resistance of TPRS decreases with the pressure and that the TPRS exhibits high resistance sensitivity to the pressure.

Different pressure points under the foot can reflect gait information. If the force points of the TPRS are not properly selected, the gait information will not be detected correctly [36]. Here, we demonstrated a wireless wearable plantar pressure capturing device that employed our proposed textile piezoresistive sensor. In the fabrication of the sensor insole, six piezoresistive sensors were mounted at the main weight-bearing positions; in particular, a sensor was placed under the big toe, the first and fourth metatarsals, the midfoot, and two sensors were allocated under the heel. The proposed insole was configured to collect and save the data in real time whilst walking at a comfortable speed. A dedicated electronic control module comprising a microcontroller, an acquisition circuit, and a battery was mounted on the front of the shoe, as shown in Figure 1g. The functional structure of the control module is given in Figure S3. During the walking trials, the captured TPRS data were transferred in real time to a dedicated wireless monitoring system using a Bluetooth Low Energy v. 5 communication channel. The wireless monitoring system allowed remote monitoring of the pressure sensor signals, preventing the use of electrical wires that could restrict natural body movements.

**Figure 6:**
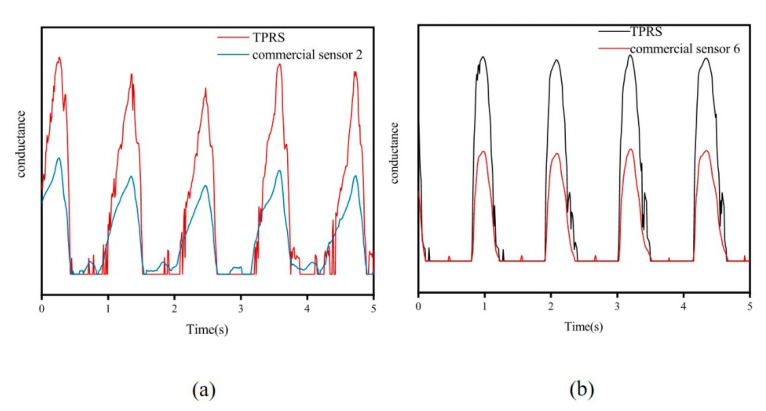
(**a**) commercial sensor placed over TPRS; (**b**) signals of sensor 2 and TPRS.

A commercial force sensor has been introduced for comparison with TPRS during walking, as shown in Figure 1f. To ensure the reference sensors and TPRS will be subjected to the same pressure during walking, the two types of sensors were placed at the same point, where the reference sensor was allocated on the upper surface of the TPRS. As shown in Figure 6, the TPRS allowed to accurately reflect the pressure pattern during walking.

**Figure 7:**
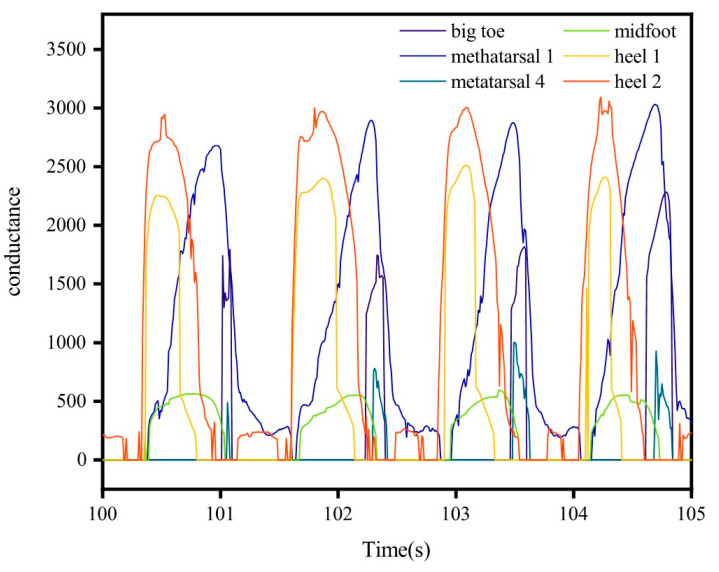
Gait cycles reflected by the signals of the designed insole.

In addition, for verifying the correct response of TPRSs integrated into the smart insole to external pressures, preparatory procedures were carried out (Figure S4, Video S1). Then, to demonstrate the excellent gait pattern recording performance of the smart insole, a healthy adult volunteer with a weight of 60 kg was invited to wear it. The volunteer wore comfortable sportswear and athletic shoes equipped with pressure-sensitive foot insole and was asked to walk over 100 m at a self-selected speed for a few minutes to get familiar with the equipment. The volunteer was then asked to perform five trials of level walking at a self-selected low speed and another five trials at a self-selected normal speed. Plantar pressure data were collected during the trials. Figure 7 shows the synchronous conductance changes of the TPRSs located in the different parts of the insole during subsequent gait cycles.

**Figure 8:**
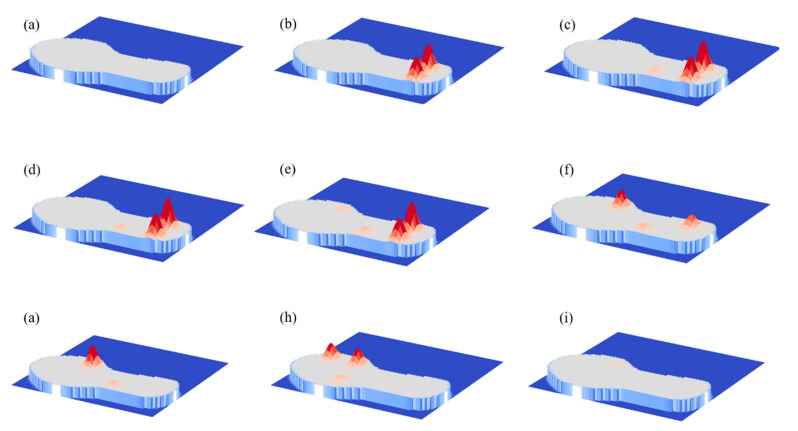
Pressure maps under the foot at different gait phases: (**a**) foot off the ground, (**b**–**d**) foot starts landing, (**e**–**f**) foot flat contact, (**g**–**h**) toe-off (**i**) foot off the ground.

The ground contact is initiated by the heel strike and reflected by the highest amplitude of the heel sensor signals. Then, the foot flat phase is reflected by the relatively low-amplitude signal of the midfoot sensor. Finally, the signals of the metatarsal sensors and the big toe sensor denote the end of the ground contact. Pressure maps are an intuitive way to understand the information of smart insole and how its sensors reflect the phases of gait. These are illustrated in Figure 8a–i (see details in supporting information Video S2). The development of the ground contact starts with the swing (Figure 8a) when pressures on all sensors are close to zero, followed by the heel contact (Figure 8b) when the heel sensor activate, midfoot (Figure 8c), first and fourth metatarsals (Figure 8e–h), toe-off (Figure 8f) and swing (Figure 8i), respectively. Judging from the experimental result, the pressure under the fourth metatarsal was higher than other parts during walking. In contrast, the sensor at the midfoot received pressure with lowest amplitude. The proposed sensor may be employed to feedback gait information and to assess health status for both hemiparetic patients and healthy individuals. These results demonstrated that the proposed insole is suitable for real-time gait monitoring in various environments.

## 4. Conclusions

In summary, we have designed a novel piezoresistive sensor for pressure measurement in wearable medical devices. It takes advantage of the excellent mechanical and electrical properties of two kinds of fabric electrodes. The proposed TPRS demonstrated a high sensitivity of 3.96 kPa^−1^ in the lower pressure range of 0–36 kPa, the ability to operate in a wide pressure range (0–100 kPa), an outstanding response to external pressure, high durability (1000 cycles) and a fast response time (170 ms); these qualities confirm its potential for real-time detection of human movement. We demonstrated the application of the sensor in a smart insole for capturing plantar pressure. The insole was prepared by mounting a TPRS into six different points beneath the foot. The developed monitoring insole takes advantage of the proposed sensor device to provide stable signals at different contact pressures. The insole performs fine-synchronized capture of all TPRS sensors, allowing the derivation of time-pressure maps reflecting the gait phases and facilitating further analysis of the collected data. The proposed TPRS can potentially find applications in electronic skins and other wearable sensing applications.

## Supplementary Materials

The following are available online at www.mdpi.com/2072-666X/12/2/110/s1, Figure S1: Surface resistance of (a) rGO cotton fabric electrode; (b) Ag fabric circuit electrode. Figure S2: The sensor performance of the TPRS under different pressures. Figure S3: Block diagram of the hardware configuration. Figure S4: (a) The response of different parts of the insole to the changes of the external pressure at a certain time during walking, (b) The response of a point on the insole to stable pressure. Video S1: The proper response of the TPRSs to external pressures Video S2: Pressure distribution diagram

## References

[1] Wang S., Oh J.Y., Xu J., Tran H., Bao Z. (2018). Skin-Inspired Electronics: An Emerging Paradigm. Acc. Chem. Res..

[2] Wang Y., Li W., Zhou Y., Jiang L., Ma J., Chen S., Jerrams S., Zhou F. (2020). Fabrication of high-performance wearable strain sensors by using CNTs-coated electrospun polyurethane nanofibers. J. Mater. Sci..

[3] Xie T., Liu Q., Xue G., Gou X. (2020). Numerical analysis of piezoelectric and mechanical response of buckled poly(vinylidene fluoride) nanofibers for the design of highly stretchable electronics. J. Mater. Sci..

[4] Wu Y., Xu W., Liu J.J., Huang M.-C., Luan S., Lee Y. (2014). An energy-efficient adaptive sensing framework for gait monitoring using smart insole. IEEE Sens. J..

[5] La T.-G., Qiu S., Scott D.K., Bakhtiari R., Kuziek J.W.P., Mathewson K.E., Rieger J., Chung H. (2018). Two-Layered and Stretchable e-Textile Patches for Wearable Healthcare Electronics. Adv. Healthc. Mater..

[6] Castano L.M., Flatau A.B. (2014). Smart fabric sensors and e-textile technologies: A review. Smart Mater. Struct..

[7] Li Y., Miao X., Raji R.K. (2019). Flexible knitted sensing device for identifying knee joint motion patterns. Smart Mater. Struct..

[8] Choong C.L., Shim M.B., Lee B.S., Jeon S., Ko D.S., Kang T.H., Bae J., Lee S.H., Byun K.E., Im J. (2014). Highly stretchable resistive pressure sensors using a conductive elastomeric composite on a micropyramid array. Adv. Mater..

[9] Liu M., Pu X., Jiang C., Liu T., Huang X., Chen L., Du C., Sun J., Hu W., Wang Z.L. (2017). Large-Area All-Textile Pressure Sensors for Monitoring Human Motion and Physiological Signals. Adv. Mater..

[10] Yang T., Xie D., Li Z., Zhu H. (2017). Recent advances in wearable tactile sensors: Materials, sensing mechanisms, and device performance. Mater. Sci. Eng. R Rep..

[11] Forintos N., Czigany T. (2020). Reinforcing carbon fibers as sensors: The effect of temperature and humidity. Compos. Part A Appl. Sci. Manuf..

[12] Tung T.T., Nine M.J., Krebsz M., Pasinszki T., Coghlan C.J., Tran D.N., Losic D. (2017). Recent advances in sensing applications of graphene assemblies and their composites. Adv. Funct. Mater..

[13] Wu W., Wen X., Wang Z.L. (2013). Taxel-addressable matrix of vertical-nanowire piezotronic transistors for active and adaptive tactile imaging. Science.

[14] Zeng W., Shu L., Li Q., Chen S., Wang F., Tao X.M. (2014). Fiber-based wearable electronics: A review of materials, fabrication, devices, and applications. Adv. Mater..

[15] Gong S., Schwalb W., Wang Y., Chen Y., Tang Y., Si J., Shirinzadeh B., Cheng W. (2014). A wearable and highly sensitive pressure sensor with ultrathin gold nanowires. Nat. Commun..

[16] Lipomi D.J., Vosgueritchian M., Tee B.C., Hellstrom S.L., Lee J.A., Fox C.H., Bao Z. (2011). Skin-like pressure and strain sensors based on transparent elastic films of carbon nanotubes. Nat. Nanotechnol..

[17] Wang X., Gu Y., Xiong Z., Cui Z., Zhang T. (2014). Silk-molded flexible, ultrasensitive, and highly stable electronic skin for monitoring human physiological signals. Adv. Mater..

[18] Cao M., Wang M., Li L., Qiu H., Padhiar M.A., Yang Z. (2018). Wearable rGO-Ag NW@ cotton fiber piezoresistive sensor based on the fast charge transport channel provided by Ag nanowire. Nano Energy.

[19] Mei Z., Ivanov K., Zhao G., Li H., Wang L. (2017). An explorative investigation of functional differences in plantar center of pressure of four foot types using sample entropy method. Med Biol. Eng. Comput..

[20] Mei Z., Zhao G., Ivanov K., Guo Y., Zhu Q., Zhou Y., Wang L. (2013). Sample entropy characteristics of movement for four foot types based on plantar centre of pressure during stance phase. Biomed. Eng. Online.

[21] Pang Z., Yang G., Khedri R., Zhang Y. (2018). Introduction to the Special Section: Convergence of Automation Technology, Biomedical Engineering, and Health Informatics Toward the Healthcare 4.0. IEEE Rev. Biomed. Eng..

[22] Tao W., Liu T., Zheng R., Feng H. (2012). Gait analysis using wearable sensors. Sensors.

[23] Verghese J., Lipton R.B., Hall C.B., Kuslansky G., Katz M.J., Buschke H. (2002). Abnormality of gait as a predictor of non-Alzheimer’s dementia. N. Engl. J. Med..

[24] Urry S. (1999). Plantar pressure-measurement sensors. Meas. Sci. Technol..

[25] Adkin A.L., Frank J.S., Carpenter M.G., Peysar G.W. (2000). Postural control is scaled to level of postural threat. Gait Posture.

[26] Orlin M.N., McPoil T.G. (2000). Plantar pressure assessment. Phys. Ther..

[27] Wearing S.C., Urry S.R., Smeathers J.E. (2000). The effect of visual targeting on ground reaction force and temporospatial parameters of gait. Clin. Biomech..

[28] Winter D.A. (1991). The Biomechanics and Motor Control of Human Gait: Normal, Elderly, and Pathological.

[29] Crea S., Donati M., De Rossi S.M., Oddo C.M., Vitiello N. (2014). A wireless flexible sensorized insole for gait analysis. Sensors.

[30] Heng W., Pang G., Xu F., Huang X., Pang Z., Yang G. (2019). Flexible Insole Sensors with Stably Connected Electrodes for Gait Phase Detection. Sensors.

[31] Wang C., Kim Y., Shin H., Min S.D. (2019). Preliminary Clinical Application of Textile Insole Sensor for Hemiparetic Gait Pattern Analysis. Sensors.

[32] Park S.W., Das P.S., Park J.Y. (2018). Development of wearable and flexible insole type capacitive pressure sensor for continuous gait signal analysis. Org. Electron..

[33] Aqueveque P., Osorio R., Pastene F., Saavedra F., Pino E. (2018). Capacitive Sensors Array for Plantar Pressure Measurement Insole fabricated with Flexible PCB. Conf. Proc. IEEE Eng. Med. Biol. Soc..

[34] Ngueleu A.M., Blanchette A.K., Bouyer L., Maltais D., McFadyen B.J., Moffet H., Batcho C.S. (2019). Design and Accuracy of an Instrumented Insole Using Pressure Sensors for Step Count. Sensors.

[35] Paredes-Madrid L., Palacio C.A., Matute A., Parra Vargas C.A. (2017). Underlying Physics of Conductive Polymer Composites and Force Sensing Resistors (FSRs) under Static Loading Conditions. Sensors.

[36] Rosenbaum D., BECKER H.P. (1997). Plantar pressure distribution measurements. Technical background and clinical applications. Foot Ankle Surg..

[37] Park S., An J., Potts J.R., Velamakanni A., Murali S., Ruoff R.S. (2011). Hydrazine-reduction of graphite- and graphene oxide. Carbon.

[38] Haniff M.A.M., Hafiz S.M., Wahid K.A., Endut Z., Wah Lee H., Bien D.C., Azid I.A., Abdullah M.Z., Huang N.M., Rahman S.A. (2015). Piezoresistive effects in controllable defective HFTCVD graphene-based flexible pressure sensor. Sci. Rep..

