# Supplementary material for: A Soft Wearable and Fully-Textile Piezoresistive Sensor for Plantar Pressure Capturing
Source: Micromachines (Basel). 2021 Jan 22;12(2):0. doi: 10.3390/mi12020110 (PMC7926843; doi:10.3390/mi12020110)
Supplement: Supplementary file 1 [file micromachines-12-00110-s001.zip › Supporting Informatin/Supporting Informatin/micromachines-1059581-supplementary.docx]

A Soft Wearable and Full Textile Piezoresistive Sensor for Plantar Pressure Capturing

Yongsong Tan ^1,2 #^, Kamen Ivanov^1,3 #^, Zhanyong Mei^4^, Hui Li^1^, Huihui Li^1^, Ludwig Lubich^5^, Chaoxia Wang ^2 *^, Lei Wang ^1 *^


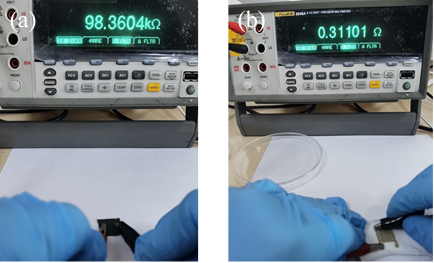


**Figure S1.** Surface resistance of (**a**) rGO cotton fabric electrode; (**b**) Ag fabric circuit electrode.

**
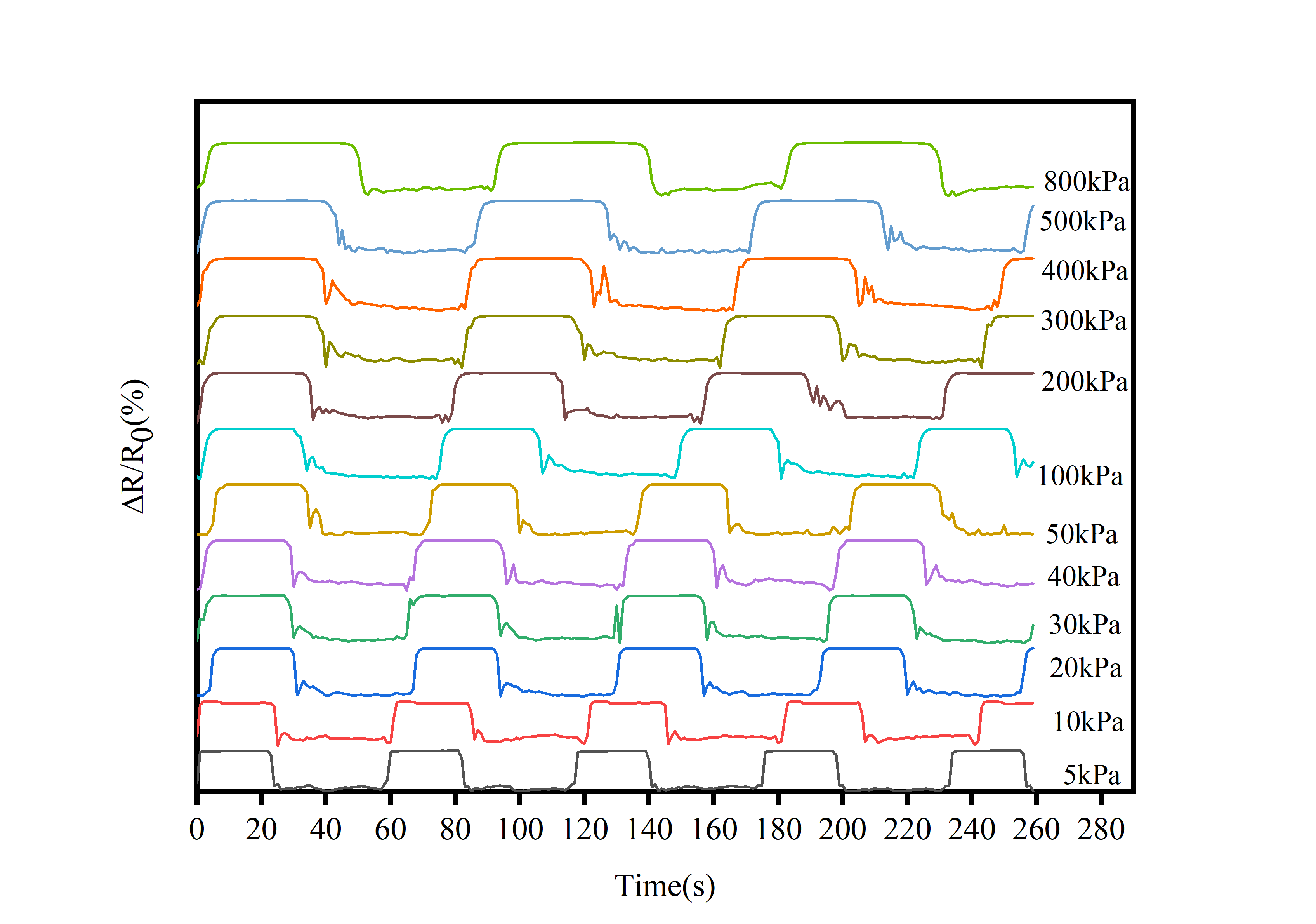
**

**Figure S2.** The sensor performace of the TPRS on different pressure.


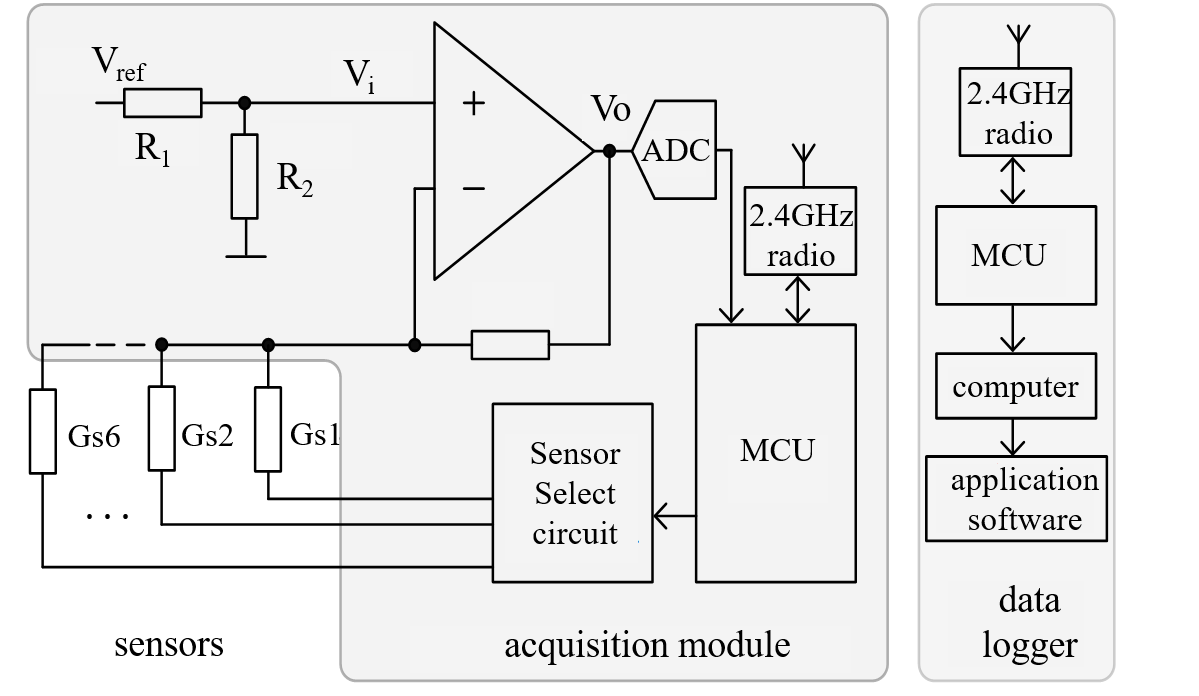


**Figure S3.** Block diagram of the full hardware configuration.

To obtain the signals of all insole sensors in real time, it was appropriate to apply and adapt a previously developed acquisition module. The block diagram of the sensor is given in Fig. 2.The microprocessor at the sensor side is integrated into the system-on-chip nRF52832 and was responsible for serving all tasks, including controlling the sensor selection circuit, reading the analog-to-digital converter and transmitting the sensor data over a Bluetooth Low Energy interface to the host computer, where received data were visualized and recorded. During the experiment, error-free transmission of data was achieved thanks to maintaining a reasonably close distance between the insole and data logger and using continuous checks of received data, as well as an external antenna at the logger side that extends its range.

The microcontroller at the sensor side scans the sensors sequentially in time, where the individual sampling frequency of each sensor is 100 Hz, a rate that can reflect enough signal changes observed during walking. The currently read sensor is selected through control logic for sensor selection.

The interface circuit based on a transimpedance amplifier operates under the presumption that the current through the sensor changes linearly with the applied force. In that:

$V_{i}=V_{ref}\frac{R2}{R1+R2}=\sigma V_{ref}$ (1)

Thanks to the fact that Vref acts as a reference of the analog-to-digital converter, the sensor conductance is derived directly as:

$Gs=\frac{\left( \frac{ADCreading}{ADClevels}-\sigma\right)}{\sigma Rf}$ (2)

In the interface circuit, we had to adjust the feedback resistor Rf to match the range of the proposed sensor.


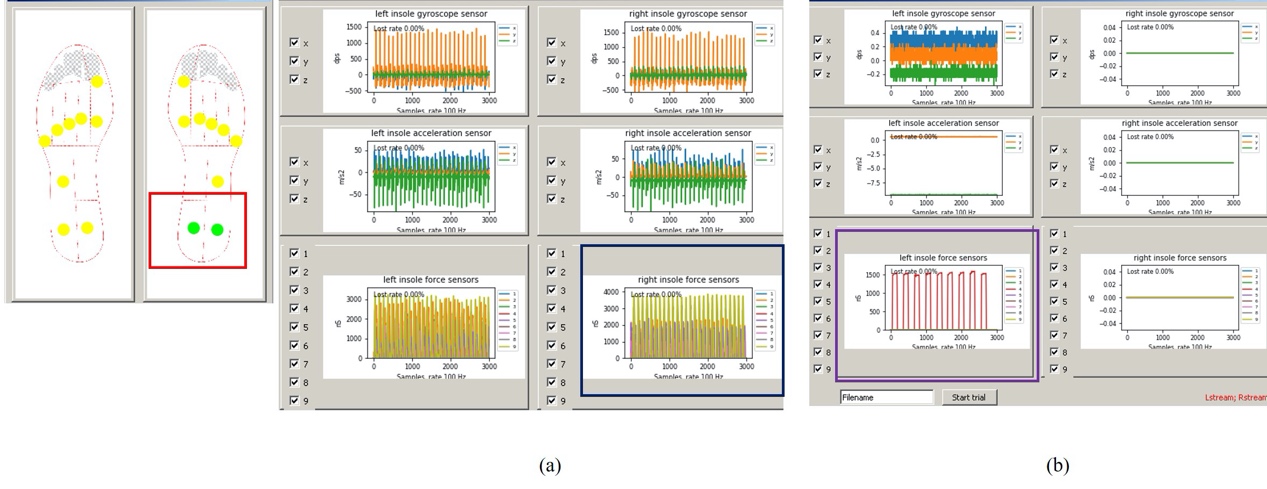


**Figure S4.** (**a**) The response of different parts of insole to the change of external pressure at a certain time during walking, (**b**) the response of a point on the insole to stable pressure.

The testing process has been conducted, as shown in Fig S3a,b. When heel 1 and heel 2 received external pressure, the light at the left insole part of Fig S3a will be on and the yellow part will show the change of sensor resistance. And the TPRS can also make a sensitive and stable response when a cyclic fixed-point force is applied to a certain part of the insole, as shown in the purple area of Fig 3b. After testing each part of the insole, the results show that the insole can respond sensitively to the external pressure changes. Through observing the reaction of insole sensor to external pressure, we can see that the insole can reflect the change of sole pressure accurately and quickly in real time.
